# Influenza A H1N1 Pandemic Strain Evolution – Divergence and the Potential for Antigenic Drift Variants

**DOI:** 10.1371/journal.pone.0093632

**Published:** 2014-04-03

**Authors:** Eili Y. Klein, Adrian W. R. Serohijos, Jeong-Mo Choi, Eugene I. Shakhnovich, Andrew Pekosz

**Affiliations:** 1 Center for Advanced Modeling in the Social, Behavioral, and Health Sciences, Department of Emergency Medicine, Johns Hopkins School of Medicine, Baltimore, Maryland, United States of America; 2 Center for Disease Dynamics, Economics, and Policy, Washington, DC, United States of America; 3 Department of Chemistry and Chemical Biology, Harvard University, Cambridge, Massachusetts, United States of America; 4 W. Harry Feinstone Department of Molecular Microbiology and Immunology, Johns Hopkins University, Bloomberg School of Public Health, Baltimore, Maryland, United States of America; National Institutes of Health, United States of America

## Abstract

The emergence of a novel A(H1N1) strain in 2009 was the first influenza pandemic of the genomic age, and unprecedented surveillance of the virus provides the opportunity to better understand the evolution of influenza. We examined changes in the nucleotide coding regions and the amino acid sequences of the hemagglutinin (HA), neuraminidase (NA), and nucleoprotein (NP) segments of the A(H1N1)pdm09 strain using publicly available data. We calculated the nucleotide and amino acid hamming distance from the vaccine strain A/California/07/2009 for each sequence. We also estimated *P_epitope_*–a measure of antigenic diversity based on changes in the epitope regions–for each isolate. Finally, we compared our results to A(H3N2) strains collected over the same period. Our analysis found that the mean hamming distance for the HA protein of the A(H1N1)pdm09 strain increased from 3.6 (standard deviation [SD]: 1.3) in 2009 to 11.7 (SD: 1.0) in 2013, while the mean hamming distance in the coding region increased from 7.4 (SD: 2.2) in 2009 to 28.3 (SD: 2.1) in 2013. These trends are broadly similar to the rate of mutation in H3N2 over the same time period. However, in contrast to H3N2 strains, the rate of mutation accumulation has slowed in recent years. Our results are notable because, over the course of the study, mutation rates in H3N2 similar to that seen with A(H1N1)pdm09 led to the emergence of two antigenic drift variants. However, while there has been an H1N1 epidemic in North America this season, evidence to date indicates the vaccine is still effective, suggesting the epidemic is not due to the emergence of an antigenic drift variant. Our results suggest that more research is needed to understand how viral mutations are related to vaccine effectiveness so that future vaccine choices and development can be more predictive.

## Introduction

In April 2009, a novel human influenza A(H1N1) virus was identified. This virus rapidly spread around the globe causing significant morbidity and mortality in 2009/2010. This virus was of swine origin [1], [2] and contained a novel combination of gene segments not previously reported in a human influenza virus isolate [3]. Except for the elderly, the vast majority of individuals around the world did not have protective immunity against the virus and were thus susceptible to infection [4]. This relatively low immunological pressure has presumably contributed to the fact that there has been only limited antigenic change in the virus.

The primary target of the immune response to influenza is generally the hemagglutinin (HA), a glycoprotein found on the surface of the virus. Mutations in the HA protein enable the virus to escape the neutralizing antibody response induced by vaccination or infection. Changes in the major antigenic epitopes are believed to be primarily responsible for immune escape [5], though changes outside these regions may also influence HA antigenic structure and antibody binding strength. More generally, evidence from equine and human challenge studies [6] suggest that reinfection probability increases as the number of amino acid differences between the primary infection/vaccine strain and the challenge strain increase. Studies at the household level found reinfection with human A(H3N2) occurred when the number of amino acid mutations was between 9 and 22 [7]. In vitro studies of the A(H1N1)pdm09 strain have shown that only one or two amino acid changes can reduce the ability of human sera to bind viruses of this strain [8].

Between April 2009 and April 2010, the Centers for Disease Control and Prevention (CDC) estimate that there were ∼61 million clinical cases of influenza in the US [9], and a further ∼80 million people were vaccinated against the virus [10]. Prior infection or vaccination precludes infection with a similar strain of influenza because the HA proteins displayed on the surface of the virus are targeted by existing antibodies. Immunity exerts pressure on the virus to evolve rapidly, a process of antigenic change well described in prior influenza epidemics. However, despite the potential for antigenic changes in the virus that may presage the emergence of an antigenic drift variant, no quantification of the magnitude of changes in the HA gene of the A(H1N1)pdm09 strain has been done on a global level. While geographically limited assessments have shown changes in the sequence of the HA gene [11]–[14], a global perspective is necessary because new strains can spread around the globe in months or even weeks. In this report we explore the evolution of the A(H1N1)pdm09 strain since April 2009 at both the RNA and protein levels, altogether constituting >9,000 sequences of A(H1N1)pdm09.

## Methods

Both the nucleotide and amino acid sequences of the coding regions and the sequences of the hemagglutinin (HA), neuraminidase (NA), and nucleoprotein (NP) segment coding regions were obtained from the National Centre for Biotechnology Information (NCBI) influenza virus resource [15]. Full-length sequences were selected for all A/H1N1 samples collected from humans from 1/1/2009 through 12/31/2013. Multiple sequence alignment was calculated using MAFFT [16], [17] with the FFT-NS-2 progressive alignment algorithm. The multiple sequence alignment was viewed with ClustalX [18].

Sequences were then compared base-pair by base-pair (nucleotides) and amino-acid by amino-acid (proteins) with the vaccine strain (A/California/07/2009). While other options for measuring pairwise distances are possible, we used the simplest metric, called the Hamming distance. This metric assigns a zero or one depending on whether two nucleotides or amino acids are identical and has been widely used to cluster different influenza strains [19]. We then defined the distance between two sequences as the sum of the pairwise distances between their composite nucleotides or amino acids. Divergence from the vaccine strain was then calculated as the percentage of the sequence that was identical to the vaccine strain.

Percentage divergence was used to identify the pandemic strains using a relatedness criterion. After examining the way that the different isolates clustered (see Figures S1, S2, S3, S4, S5 and S6), strains with a similarity greater than a specific percentage were considered pandemic strains and all subsequent analysis was on these remaining strains. For HA and NA sequences, strains with a relatedness greater than 90% to the vaccine strain were considered A(H1N1)pdm09 strains, while for NP sequences we used 94% as the cutoff. The pandemic strains were then sorted by collection date. Strains with only the year of collection were excluded. Strains that had year and month but not day were sorted at the end of each month.

We then plotted the hamming distance of both the nucleotide coding regions and the amino acids and calculated the rate of mutation accumulation as the linear trend of the fit of the data. Strains were also separated into two seasons per year, from April to September and from October to March, and differences in the mean hamming distance between seasons were tested for statistical significance using a two-tailed student’s T-test. Linear trend analysis and significance tests were done in R [20].

### Epitope Analysis

Antibodies bind influenza virus primarily at the epitope regions of the hemagglutinin protein [21]. Although other residues can affect the geometry at the surface, and so can be under selective pressure, they are not available for presentation to antibodies. Thus, these epitopes are likely to be the predominant sites of selection and increased change in those sites is suggestive of immune escape. In addition, there have been suggestions of a linear correlation between vaccine efficacy and the antigenic distance of a strain at the epitopes from the vaccine strain [22].

Despite the importance of the epitope regions there is no consensus on the epitope regions for A(H1N1)pdm09. We thus examined three different possible models suggested in the literature. The first was done by Deem *et al.* [5], which mapped five epitope regions (A-E) from H3 onto a pandemic strain (A/California/04/2009). The second one we used was proposed by Huang et al. [23] and uses entropy and a likelihood ratio to define a set of 41 natural epitopes that are a subset of the five epitope regions defined by Deem *et al*. [5]. The third is the set of five antigenic regions (Ca1, Ca2, Cb, Sa, Sb) defined from laboratory studies on influenza virus A/PR/8/34 [24]. For each set of epitope regions, we calculated the hamming distance for each region as well as *P*_epitope_, a measure of antigenic distance [25] defined as,
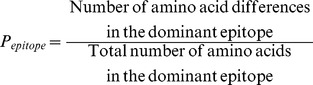
(1)which can be used to estimate the likely efficacy of a vaccine [22], [25]. We also analyzed the rate of non-synonymous to synonymous (dN/dS) changes in the coding region of the HA gene across all the isolates using the vaccine strain as the basis for comparison, focusing on the differences in the rates between epitope (using the first definition) and non-epitope residues.

### H3N2

We also conducted a similar analysis comparing changes in the HA gene between A(H3N2) strains and the H3N2 vaccine strains. Full-length H3N2 sequences were also downloaded from the NCBI influenza virus resource [15] for the period 1/1/2009 through 12/31/2013. Multiple sequence alignment was again calculated using MAFFT [16], [17] with the FFT-NS-2 progressive alignment algorithm, and the multiple sequence alignment was viewed with ClustalX [18]. Finally, base-pair by base-pair (nucleotides) and amino-acid by amino-acid (proteins) comparison was done with the vaccine strain for each season as noted by the WHO (http://www.who.int/influenza/vaccines/virus/recommendations/en/). Thus, strains collected prior to April 2010, were compared to the A/Brisbane/10/2007 strain. Strains collected between April 2010 and October 2012 were compared to A/Perth/16/2009, and strains collected after October 2012 were compared to vaccine strain A/Victoria/361/2011.

## Results

We calculated the hamming distance for both the coding region and the protein of the HA, NA, and NP gene segments for all available fully sequenced strains of A(H1N1)pdm09 in the NCBI influenza virus resource from April 1999 to December 2013. The total number of HA sequences was 9,076 (includes one that was dated March 30, 2009 but not the vaccine strain), though sampling was not equal across the years, with the vast majority (75%) sequenced between April 2009 and March 2010 (Table 1). There were fewer fully sequenced NA and NP isolates, only 7,232 and 4,406, respectively. Despite these limitations, clear trends were observed in the rate that the HA, NA, and NP genes and proteins accumulated mutations.

**Table 1:** Mean hamming distance (standard deviation) of the hemagglutinin, neuraminidase and nucleoprotein coding regions and proteins, by season.

	4/2009–9/2009*	10/2009–3/2010	4/2010–9/2010	10/2010–3/2011	4/2011–9/2011	10/2011–3/2012	4/2012–9/2012	10/2012–3/2013	4/2013–9/2013†	10/2013–12/2013†
**A(H1N1)pdm09 RNA Coding Region**										
**HA**	7.4 (2.2)	10.0 (2.0)	13.0 (2.4)	17.3 (3.5)	20.5 (3.2)	24.8 (7.3)	27.7 (12.7)	26.0 (4.5)	27.7 (2.4)	28.3 (2.1)
**NA**	3.7 (1.4)	5.5 (1.8)	8.1 (3.0)	10.8 (6.3)	12.0 (2.7)	14.9 (2.3)	14.5 (2.5)	14.3 (3.1)	14.9 (1.8)	
**NP**	5.2 (3.0)	7.2 (1.7)	9.7 (1.7)	10.1 (1.9)	10.9 (2.9)	13.2 (9.5)	14.0 (0.0)	20.1 (4.3)		
**A(H1N1)pdm09 Protein**										
**HA**	3.6 (1.3)	4.8 (1.4)	6.6 (1.4)	8.4 (1.6)	9.3 (1.8)	11.7 (2.4)	12.0 (3.8)	11.1 (2.1)	12.0 (1.2)	11.7 (1.0)
**NA**	2.0 (0.9)	2.8 (1.0)	4.0 (1.2)	5.2 (2.3)	6.1 (2.2)	6.4 (1.3)	6.2 (1.4)	6.1 (1.6)	8.0 (1.9)	
**NP**	3.1 (0.6)	3.2 (0.6)	3.7 (0.8)	3.4 (0.6)	3.5 (0.9)	3.6 (1.4)	3.0‡	4.0 (0.6)		
**A(H1N1)pdm09 Number of Sequences**										
**HA**	4014	2811	418	951	107	238	63	391	67	43
**NA**	3150	2198	372	845	49	206	102	264	45	0
**NP**	2381	1316	196	299	15	53	2	142	0	0
**H3N2**										
**HA RNA Coding Region**	18.1 (6.4)	21.2 (2.4)	19.5 (5.4)	23.9 (5.7)	25.9 (15.0)	29.8 (22.3)	28.7 (4.3)	21.7 (9.4)	19.1 (3.2)	19.5 (1.3)
**HA Protein**	7.3 (3.3)	8.9 (1.6)	7.6 (1.5)	8.7 (1.8)	10.1 (5.5)	11.5 (8.3)	11.2 (1.9)	10.1 (3.8)	9.8 (1.6)	9.5 (1.7)
**Number of Sequences**	582	47	209	684	286	471	198	685	57	4

*Includes a sequence dated 3/30/2009.

† No NP/NA sequences were uploaded to the database for this period.

‡ Too few samples to calculate standard deviation.

**Figure 1:**
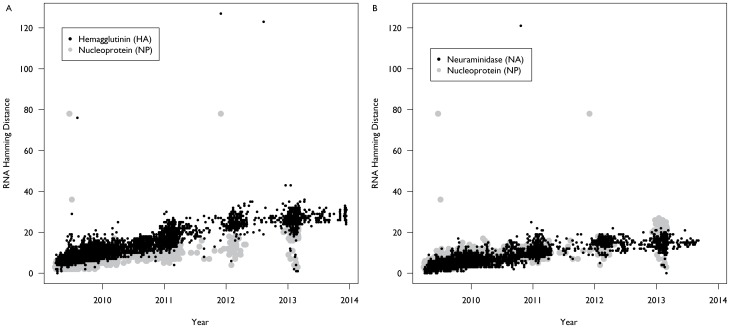
RNA Divergence of Influenza H1N1 Pandemic Strain, 2009–2013. Since April 2009, the coding region of the hemagglutinin segment of the influenza H1N1 pandemic strain has been accumulating nucleotide mutations at a rate of approximately 5.68 mutations per annum (A). This contrasts with the coding regions for the neuraminidase and nucleoprotein segments which have been accumulating mutations at a rate of only 3.56 and 3.81 mutations per annum, respectively (B).

Between April 2009 and December 2013, the coding region of the hemagglutinin segment of the influenza H1N1 pandemic strain accumulated nucleotide mutations at a faster rate than the coding regions of the NA and NP segments (Figure 1). We estimated that the HA gene has been accumulating mutations at a rate of approximately 5.68 (Standard Error [SE]: 0.03) mutations per annum, or a rate of 3.3×10^−3^ nucleotide substitutions per site per year. This contrasts with the coding regions for the neuraminidase and nucleoprotein segments which have been accumulating mutations at a rate of only 3.56 (SE: 0.04) and 3.81 (SE: 0.05) mutations per annum, respectively, which is 2.5×10^−3^ nucleotide substitutions per site per year for both.

The average hamming distance from the vaccine strain in the RNA coding region for the HA gene increased from 7.4 (standard deviation [SD]: 2.2) for strains collected in the initial season between April 2009 and September 2009 to 24.8 (SD: 7.3) for the 2011–2012 northern hemisphere influenza season (Table 1). Each year’s increase was statistically significant (p<0.01) both compared to the prior season as well as the initial season. However, while the following seasons were statistically different from the initial season (p<0.01), the mean hamming distance has not significantly changed since March 2012. This is also reflected in the mutation accumulation rate, which was 6.54 (SE: 0.04) per annum, or a rate of 3.8×10^−3^ nucleotide substitutions per site per year, for the HA gene between April 2009 and March 2012.

**Figure 2:**
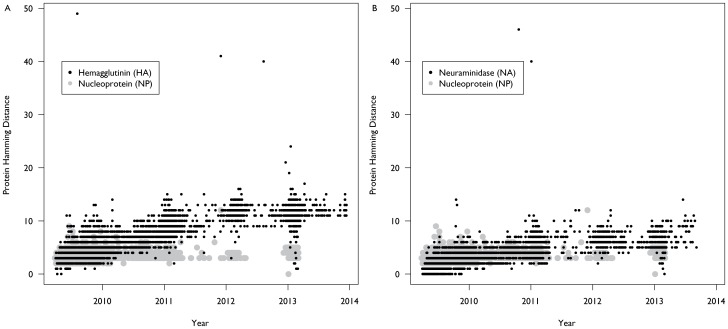
Protein Divergence of Influenza H1N1 Pandemic Strain, 2009–2013. Since April 2009, the hemagglutinin protein of the influenza H1N1 pandemic strain has been accumulating mutations at a rate of approximately 2.45 mutations per annum (A). This contrasts with the neuraminidase and nucleoprotein proteins which have been accumulating mutations at a rate of only 1.52 and 0.26 mutations per annum, respectively (B). In addition, there were more outliers present in 2013, which suggests that the population that was infected was smaller, presenting opportunities for increasing diversity.

The hemagglutinin protein of the influenza H1N1 pandemic strain has also been accumulating mutations at a faster rate than the NA and NP proteins (Figure 2). We estimated that the HA protein has been accumulating mutations at a rate of approximately 2.45 (SE: 0.02) mutations per annum, or 4.3×10^−3^ amino acid substitutions per site per year, while the neuraminidase and nucleoprotein proteins have been accumulating mutations at a rate of only 1.52 (SE: 0.02) and 0.26 (SE: 0.01) mutations per annum, or a rate of 3.2×10^−3^ and 0.51×10^−3^ amino acid substitutions per-site per-year, respectively. Average hamming distance for the HA protein from the vaccine strain increased from a mean of 3.6 (SD: 1.4) for strains collected between April 2009 and September 2009 to 11.7 (SD: 2.4) for strains collected between October 2011 and March 2012 (Table 1). Each year’s increase was also statistically significant (p<0.01) both compared to the prior season as well as the initial season. However, while again the 2012–2013 seasons were statistically different from the 2009–2010 season (p<0.01), the mean hamming distance of the HA protein from the vaccine has not significantly changed since March 2012.

While the last two seasons have not seen significant changes in the mean hamming distance, this belies differences in the pattern of mutations between seasons. For instance, the mutation D97N fluctuates in frequency, though never reaching 50%, through several seasons before becoming dominant in 10/2012–3/2013 season. On the other hand mutations S69T, S143G, A197T, N260D, and V520A all became the consensus mutation in the 10/2011–3/2012 influenza season, appearing in ∼70–80% of sequences, but by the next year they all became much less common and the dominant amino acid found is the wild type (Table 2). A number of other mutations – P83S, S203T, and I321V – were found in most sequences by the winter of 2009 and have not waned in frequency. While, some mutations, S185T, E374K, and S451N, continually increase in frequency each season, other mutations (K163Q, K283E, A256T, and E499K) all became the dominant sequence in 10/2013–3/2013 or later for the first time after persisting at a low frequency for a number of seasons. All the mutations described here were originally seen in at least one sequence in 2009–2010, though this is not surprising as nearly 70% of the amino acids have at least one mutation in one isolate in the 10/2009–3/2010 season.

**Table 2:** Selected Mutations in the HA region of influenza A(H1N1)pdm09.

	Season									
Mutation	4/2009–9/2009†	10/2009–3/2010	4/2010–9/2010	10/2010–3/2011	4/2011–9/2011	10/2011–3/2012	4/2012–9/2012	10/2012–3/2013	4/2013–9/2013	10/2013–12/2013
S69T*	S (100%)	S (99%)	S (100%)	S (100%)	S (100%)	T (71%)	T (68%)	S (98%)	S (100%)	S (100%)
P83S*	S (99%)	S (99%)	S (99%)	S (99%)	S (99%)	S (99%)	S (100%)	S (98%)	S (100%)	S (100%)
D97N	D (99%)	D (92%)	D (94%)	D (65%)	D (54%)	D (84%)	D (75%)	N (84%)	N (96%)	N (100%)
S143G*	S (100%)	S (100%)	S (99%)	S (77%)	S (68%)	G (82%)	G (75%)	S (86%)	S (96%)	S (100%)
K163Q	K (100%)	K (99%)	K (98%)	K (96%)	K (94%)	K (99%)	K (92%)	K (76%)	Q (64%)	Q (100%)
S185T*	S (99%)	S (99%)	S (84%)	T (68%)	T (76%)	T (89%)	T (98%)	T (96%)	T (100%)	T (100%)
A197T	A (99%)	A (96%)	A (90%)	A (62%)	A (55%)	T (82%)	T (75%)	A (86%)	A (96%)	A (100%)
S203T	T (67%)	T (96%)	T (100%)	T (100%)	T (100%)	T (99%)	T (98%)	T (98%)	T (100%)	T (100%)
V234I	V (100%)	V (99%)	V (100%)	V (99%)	V (100%)	V (100%)	V (98%)	I (70%)	V (69%)	V (100%)
A256T	A (100%)	A (99%)	A (100%)	A (100%)	A (100%)	A (99%)	A (100%)	A (91%)	T (66%)	T (100%)
N260D*	N (99%)	N (100%)	N (99%)	N (99%)	N (96%)	D (71%)	D (68%)	N (98%)	N (100%)	N (100%)
K283E*	K (99%)	K (98%)	K (100%)	K (97%)	K (98%)	K (98%)	K (95%)	E (73%)	E (93%)	E (100%)
I321V	V (98%)	V (94%)	V (100%)	V (99%)	V (98%)	V (98%)	V (95%)	V (97%)	V (99%)	V (95%)
E374K	E (97%)	E (62%)	K (79%)	K (84%)	K (98%)	K (98%)	K (98%)	K (98%)	K (100%)	K (100%)
S451N	S (99%)	S (99%)	S (83%)	N (69%)	N (77%)	N (88%)	N (95%)	N (97%)	N (99%)	N (100%)
E499K	E (100%)	E (99%)	E (93%)	E (89%)	E (85%)	E (92%)	E (90%)	K (84%)	K (97%)	K (100%)
V520A	V (100%)	V (98%)	V (97%)	V (99%)	V (99%)	A (72%)	A (68%)	V (88%)	V (96%)	V (100%)

Percentage refers to the percent of sequences in that season that have the noted amino acid.

† Includes isolate collected 3/30/2009.

*Epitope residueβ.

### Epitopes

**Figure 3:**
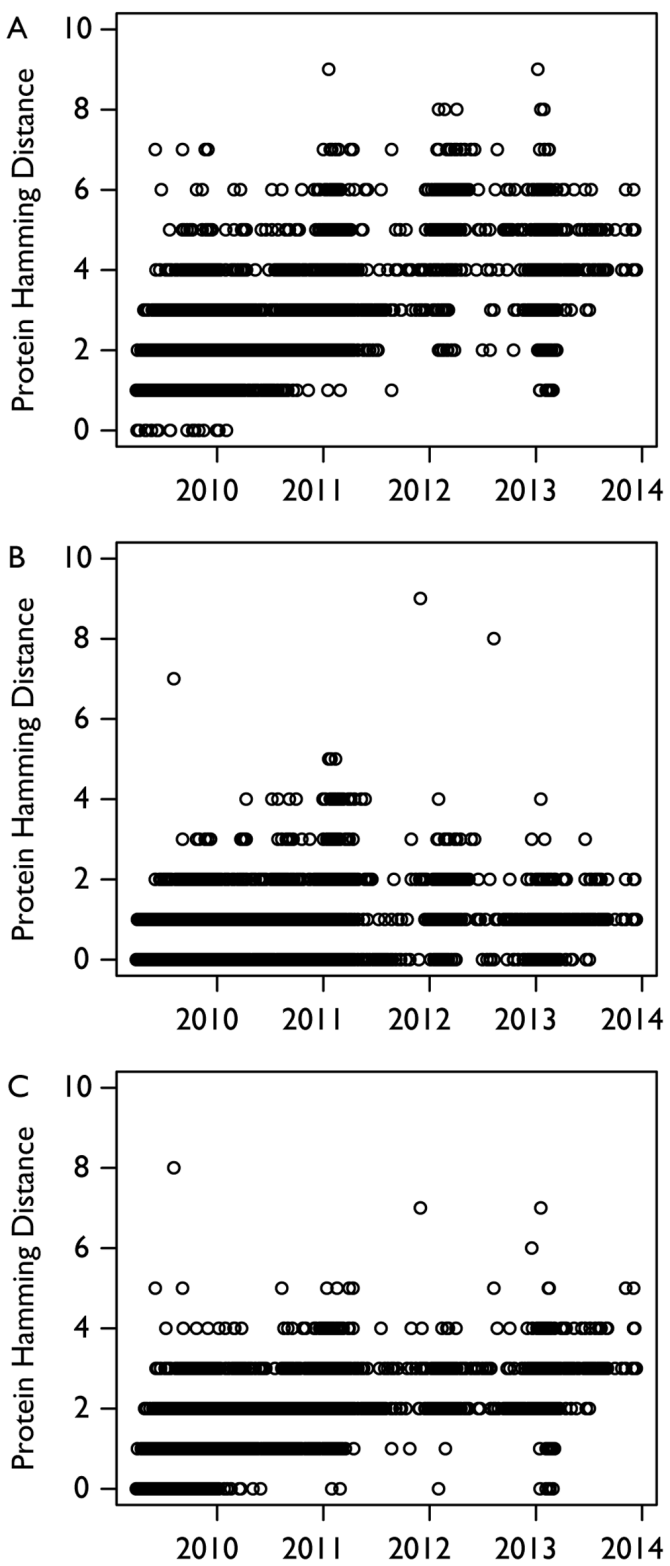
Divergence at the A(H1N1)pdm09 epitopes. Changes in the major antigenic epitopes are believed to be primarily responsible for immune escape. We examined the total number of mutations in these regions combined. However, there is disagreement as to the amino acid locations encoding the epitope regions, thus we used three potential descriptions of the epitope regions of the influenza A(H1N1)pdm09 HA protein. The first (A) was based on the A(H3N2) strain’s epitopes, the second (B) was a set of natural epitopes that is a subset of the first set of epitopes, while the third (C) is a set of laboratory confirmed epitopes for prior H1N1 strains. All three show divergence (i.e. an increase in the number of hamming distance) in the epitope regions, particularly the first and third definitions.

Because of the uncertainty regarding the location of the epitope regions of the hemagglutinin protein, we examined mutations using three different definitions for these regions: (1) a set of epitopes defined by matching the epitopes to H3N2 [5]; (2) a subset of the first set that are natural epitopes [23]; and (3) a set of laboratory confirmed sites for prior seasonal H1N1 strains [24]. In the first set, which encompasses the largest number of residues, there are persistent mutational changes in epitopes B-E (Figure S7), which results in an average of between 4 and 6 mutations in the epitope regions by the 2011–2012 influenza season (Figure 3). However as the mutations are spread around the epitopes, the average *P*_epitope_ (which measures the proportion of changes in the dominant epitope) is only 0.06 and 0.05 for the 10/2012–3/2013 and the 4/2013–9/2013 seasons, respectively, though the max *P*_epitope_ during this period is 0.27. Using only the subset of those residues which have been defined as natural epitopes we observed fewer mutations in these residues, with the majority appearing in epitope D (Figure S8). However, with a lower denominator, average *P*_epitope_ calculated for these residues is 0.08 and 0.14 for the 10/2012–3/2013 and the 4/2013–9/2013 seasons, respectively, and a maximum value of 0.3. Lastly, for the laboratory confirmed epitopes we observed that there were approximately 3 mutations in these residues on average in recent seasons, primarily in the Ca1, Sa, and Sb regions (Figure S9). This resulted in a *P*_epitope_ average value of 0.11 in both the 10/2012–3/2013 and the 4/2013–9/2013 seasons, and a maximum value of 0.42.

Analysis of the non-synonymous to synonymous mutations in the epitope regions compared to the rest of the gene found that dN/dS outside the epitope regions was fairly high in the first couple seasons but has been approximately one in the last several seasons. Conversely, within the epitope regions dN/dS has generally been above unity (Figure S10).

### H3N2

**Figure 4:**
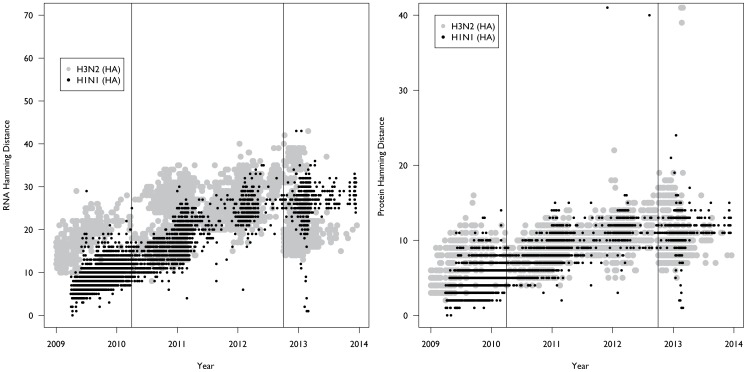
Comparison of A(H1N1)pdm09 and A(H3N2) evolution in the Hemagglutinin gene. We measured the hamming distance of the nucleotides (A) and the amino acids (B) relative to the vaccine strain for the particular season. For H1N1 the vaccine strain (A/California/07/2009) has not changed since 2009, though it was not administered until November 2009. For H3N2 the vaccine strain was changed twice over the study (where the vertical lines are). Thus, strains collected prior to April 2010, were compared to the A/Brisbane/10/2007 strain. Strains collected between April 2010 and October 2012 were compared to A/Perth/16/2009, and strains collected after October 2012 were compared to vaccine strain A/Victoria/361/2011.

Annual influenza epidemics in the United States in the 2010–2011, 2011–2012, and 2012–2013 seasons were predominated by H3N2 influenza strains (http://www.cdc.gov/flu/weekly/pastreports.htm). We calculated the hamming distance for both the coding region and the protein of the HA gene segments for all available fully sequenced isolates of A(H3N2) in the NCBI influenza virus resource from January 1999 to December 2013. The total number of HA sequences was 3,220, and sampling was approximately equal across years. We then compared the number of mutations that differed between collected strains in each season with the recommended vaccine strain for that season, and compared this to the evolution of A(H1N1)pdm09 over the period of the study. This data indicates that H3N2 mutation rates similar to that seen with A(H1N1)pdm09 led to the emergence of two antigenic drift variants but no A(H1N1)pdm09 drift variants emerged during the same timeframe (Figure 4).

## Discussion

Since the emergence of the A(H1N1)pdm09 virus in 2009, only a limited number of genetic or antigenic changes in the virus HA gene/protein have been documented. Based on the detected sequences, which remained antigenically homogeneous and closely related to the vaccine virus, the recommended virus strain for inclusion in the seasonal influenza vaccine remained an A/California/07/2009-like virus for the 2013–2014 northern hemisphere winter influenza season [26] as well as for the upcoming 2014 southern hemisphere influenza season [27] and the 2014–2015 northern hemisphere winter influenza season. However, over the past several seasons there have been a number of reports of virus isolates containing amino acid changes in the HA protein that have the potential to alter the antigenic properties of the virus [8], [28]. In this report we observe that the HA protein has accumulated mutations both in total and within the epitope regions that make the potential for vaccine escape highly probable. This has important implications for evolutionary, epidemiological, and clinical aspects of the virus.

From an evolutionary perspective, the HA gene has been accumulating mutations more rapidly than the NA and NP genes, however, the rate of nucleotide substitution and amino acid substitution is lower than prior estimates [29]. While the faster mutational drift of the HA gene is similar to past experience with other H1N1 strains as well as with H3N2 strains, the slower rate of mutation is consistent with the theory that because most individuals born after 1957 were susceptible to the virus, immune pressure should have been relatively weaker, slowing the rate that the virus evolved. Surprisingly though, we observed that the vast majority of the mutations occurred during the 2009 to 2012 time period, with only limited changes occurring over the last year. This contrasts with H3N2 which has seen a continual increase in mutational difference (Figure S11). This suggests that the two viruses may be subject to different selective pressures on their corresponding HA proteins.

Historically, for a new epidemic to occur, the HA protein of the virus has to mutate enough to become antigenically distinct to a significant percentage of individuals [30], [31]. Prior studies suggest that the probability that this will occur increases when the number of amino acid substitutions in the HA protein exceeds 10 [6], [7] or the number of amino acid substitutions in the epitope regions exceeds 4 [29]. This is also the pattern that we see with the A(H3N2) data. Clinically the 2013–2014 season has so far been marked by an influenza epidemic predominated by A(H1N1)pdm09 [32]. This is consistent with the appearance of prior epidemics given that the number of mutations in the HA protein (particularly in the epitope regions) was similar in magnitude to prior H1N1/H3N2 epidemics that were the result of antigenic drift. However, while the estimated *P*_epitope_ scores suggested that the vaccine may be only moderately effective [22], [33], the evidence to date indicates that the vaccine for the 2013–2014 season has been as effective as prior seasons [34], [35]. The plateau in the hamming distance and the efficacy of the vaccine suggests that an antigenic drift variant has not emerged this season, despite an increase in the number of cases consistent with an epidemic. These results could be explained by several different reasons. The first is that potentially the vaccine does not provide long-lasting immunity as a natural infection would and individuals vaccinated in prior years are susceptible if they did not get a vaccine this year. A second possibility is that because the A(H1N1)pdm09 strain was a novel strain almost everyone was susceptible, but many individuals may not have been infected during the initial wave of infection leaving a large pool of susceptible individuals that has been augmented with births of naïve children. Third, the mid-season results could just be due to sampling bias and more sequences/studies may suggest an alternative narrative. The first two cases suggest that improved vaccination coverage would have contributed to fewer cases of influenza this season.

We, as yet, cannot predict how influenza mutations will accumulate or how these specific mutations will contribute to influenza epidemics. For example, over the course of the study, numerous genetic ‘outliers’ were sampled without a new epidemic occurring. In fact there were three samples in which more than 40 amino acids differed from the vaccine strain identified prior to the 2012–2013 seasons. Why did these strains not start a new epidemic? Excluding sequencing errors, one possibility is that they could have been less transmissible relative to the dominant strain and thus could not seed a new epidemic. Alternatively, as an epidemic increases and there are more infected individuals, the probability of genetic outliers appearing increases. However, as they are outliers, the probability that they are transmitted is less precisely because they are outliers (regardless of fitness – though mutations generally reduce fitness, further reducing the likelihood an outlier is selected). However, as the epidemic wanes the likelihood of a genetic outlier appearing is less, but if one is generated, the probability that it will spread is increased. This suggests that variability (i.e. genetic diversity) in sampling is likely to increase as the number of susceptible individuals wanes and the seed of a new epidemic is likely to occur from these ‘outliers’. Better predictions of how outliers are related to future epidemics could lead to an increase in the efficiency of selecting future vaccine strains.

Despite the important epidemiological and clinical implications of this work, it is not without limitations. First of all, estimates of antigenic drift and vaccine effectiveness are based in large part on changes in the epitope regions of the HA protein, however, there is no consensus on the exact location of these epitopes. In addition, the most recent H3N2 epidemic was due in large part to changes in the structure of the epitopes that occurred outside the clearly defined epitope region. Second, the samples we used were not randomly selected, but were drawn from available sequences. These sequences are largely from individuals that were hospitalized in western countries and so likely represent only a fraction of the potential diversity. Regions outside of the west may play a large role in the evolution of influenza. For instance, while the most significant outliers from 2013 were from Kenya, African isolates account for only a small fraction of the total number in the database. Better geographic surveillance would increase the potential for identifying antigenic drift in the virus and improve the capacity to make vaccine strain choices. Despite these limitations, the extremely large number of samples heralds a new era in genomic surveillance and promises to increase our knowledge and understanding as to how influenza evolves. It also suggests a need for tools to be developed that allow quick and easy interpretation of newly sequenced isolates within the context of other sequences so that decisions on surveillance and interventions can be optimally provided. Crucially it also suggests more research is needed to understand how viral mutations are related to vaccine effectiveness so that future vaccine choices can be more predictive.

## Conclusion

The vast number of A(H1N1)pdm09 sequences provides a means of understanding the evolution of influenza and potentially predicting new epidemics. Data of this sort can be used to develop theories and predictions as to how future viruses may evolve and provide data for vaccine optimization. Ideally, computational and *in vitro* methods could be used to generate vaccine strains that would be predictive rather than reactive, but a better understanding of influenza intra-host diversity and transmission is required to start developing such techniques. While the future evolutionary paths of the A(H1N1)pdm09 strain are not fully known and subject to as yet undetermined ecological and environmental effects due to interactions with other strains and pathogens, the number of mutations in the HA protein suggest that there is a high probability of an antigenic drift variant in the A(H1N1)pdm09 strain occurring in the near future, and surveillance should be geared to look for such changes.

## Supporting Information

Figure S1 **Influenza A(H1N1)pdm09 Strain Selection,** **Hemagglutinin RNA.** All H1N1 RNA coding sequences from January 1, 2009 to September 30, 2013 were compared to the H1N1 pandemic vaccine strain (A/California/07/2009) and scored for divergence based on the percentage of nucleotides that were similar at each position. The resulting clusters were then separated and non-pandemic strains – those with a divergence greater than the dashed grey line – were excluded from further analysis.
(GIF)

Figure S2 **Influenza A(H1N1)pdm09 Strain Selection,** **Hemagglutinin Protein.** All H1N1 protein sequences from January 1, 2009 to September 30, 2013 were compared to the H1N1 pandemic vaccine strain (A/California/07/2009) and scored for divergence based on the percentage of amino acids that were similar at each position. The resulting clusters were then separated and non-pandemic strains – those with a divergence greater than the dashed grey line – were excluded from further analysis.
(GIF)

Figure S3 **Influenza A(H1N1)pdm09 Strain Selection, Neuraminidase RNA.** All H1N1 RNA coding sequences from January 1, 2009 to September 30, 2013 were compared to the H1N1 pandemic vaccine strain (A/California/07/2009) and scored for divergence based on the percentage of nucleotides that were similar at each position. The resulting clusters were then separated and non-pandemic strains – those with a divergence greater than the dashed grey line – were excluded from further analysis.
(GIF)

Figure S4 **Influenza A(H1N1)pdm09 Strain Selection, Neuraminidase Protein.** All H1N1 protein sequences from January 1, 2009 to September 30, 2013 were compared to the H1N1 pandemic vaccine strain (A/California/07/2009) and scored for divergence based on the percentage of amino acids that were similar at each position. The resulting clusters were then separated and non-pandemic strains – those with a divergence greater than the dashed grey line – were excluded from further analysis.
(GIF)

Figure S5 **Influenza A(H1N1)pdm09 Strain Selection, Nucleoprotein RNA.** All H1N1 RNA coding sequences from January 1, 2009 to September 30, 2013 were compared to the H1N1 pandemic vaccine strain (A/California/07/2009) and scored for divergence based on the percentage of nucleotides that were similar at each position. The resulting clusters were then separated and non-pandemic strains – those with a divergence greater than the dashed grey line – were excluded from further analysis.
(GIF)

Figure S6 **Influenza A(H1N1)pdm09 Strain Selection, Nucleoprotein Protein.** All H1N1 protein sequences from January 1, 2009 to September 30, 2013 were compared to the H1N1 pandemic vaccine strain (A/California/07/2009) and scored for divergence based on the percentage of amino acids that were similar at each position. The resulting clusters were then separated and non-pandemic strains – those with a divergence greater than the dashed grey line – were excluded from further analysis.
(GIF)

Figure S7 **Divergence at the A(H1N1)pdm09 epitopes, definition 1.** We used three potential descriptions of the epitope regions of the influenza A(H1N1)pdm09 HA protein. The present one was based on the A(H3N2) strain’s epitopes. A-E refers to the different epitopes, while F is the *P_epitope_* calculation measuring the proportion of amino acid differences in the dominant epitope, for each strain.
(GIF)

Figure S8 **Divergence at the A(H1N1)pdm09 epitopes, definition 2.** We used three potential descriptions of the epitope regions of the influenza A(H1N1)pdm09 HA protein. The present one is a set of natural epitopes that is a subset of the first set of epitopes. A-E refers to the different epitopes, while F is the *P_epitope_* calculation measuring the proportion of amino acid differences in the dominant epitope, for each strain.
(GIF)

Figure S9 **Divergence at the A(H1N1)pdm09 epitopes, definition 3.** We used three potential descriptions of the epitope regions of the influenza A(H1N1)pdm09 HA protein. The present one is a set of laboratory confirmed epitopes for prior H1N1 strains. A-E refers to the different epitopes, Ca1, Ca2, Cb, Sa, Sb, while F is the *P_epitope_* calculation measuring the proportion of amino acid differences in the dominant epitope, for each strain.
(GIF)

Figure S10 **Non-Synonymous and Synonymous Mutations in A(H1N1)pdm09.** We calculated the ratio of non-synonymous to synonymous mutations (dN/dS) for A(H1N1)pdm09 strains relative to the vaccine strain (A/California/07/2009) for regions outside the epitope regions (A) and within the epitope regions (B) using the first definition of the epitope regions (see methods). The straight line denotes unity, which is generally considered the neutral mutation rate.
(GIF)

Figure S11 **Comparison of A(H1N1)pdm09 and A(H3N2) evolution in the Hemagglutinin gene.** We measured the hamming distance of the nucleotides (A) and the amino acids (B) relative to the vaccine strain for 2009. For H1N1 the vaccine strain (A/California/07/2009) has not changed since 2009, though it was not administered until November 2009. For H3N2 all isolates were compared to the A/Brisbane/10/2007 strain, though the vaccine has changed twice since then. While the hamming distance of H3N2 isolates from the vaccine strain continues to increase, the H1N1 isolates seem to have plateaued in recent years.
(GIF)
