# Supplementary figures and images for: Influenza A H1N1 Pandemic Strain Evolution – Divergence and the Potential for Antigenic Drift Variants
Source: PLoS One. 2014 Apr 3;9(4):e93632. doi: 10.1371/journal.pone.0093632 (PMC3974778; doi:10.1371/journal.pone.0093632)

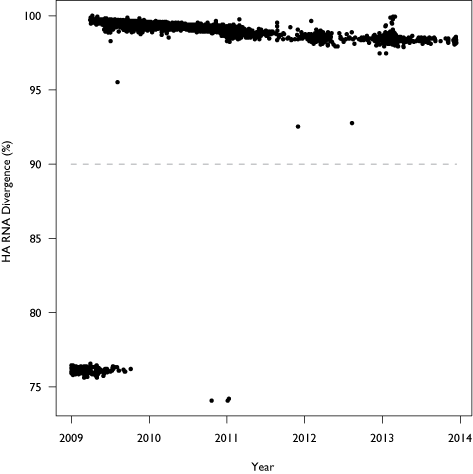

Supplement: Figure S1 — Influenza A(H1N1)pdm09 Strain Selection, Hemagglutinin RNA. All H1N1 RNA coding sequences from January 1, 2009 to September 30, 2013 were compared to the H1N1 pandemic vaccine strain (A/California/07/2009) and scored for divergence based on the percentage of nucleotides that were similar at each position. The resulting clusters were then separated and non-pandemic strains – those with a divergence greater than the dashed grey line – were excluded from further analysis. (GIF) [file pone.0093632.s001.gif]

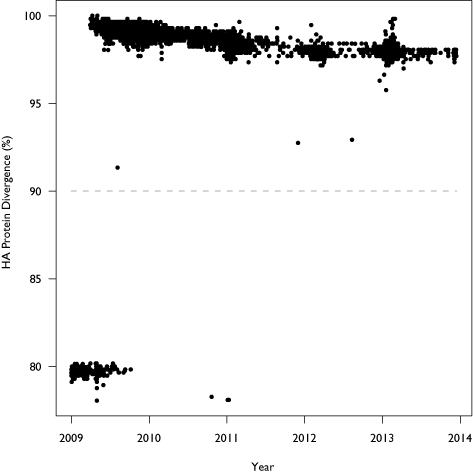

Supplement: Figure S2 — Influenza A(H1N1)pdm09 Strain Selection, Hemagglutinin Protein. All H1N1 protein sequences from January 1, 2009 to September 30, 2013 were compared to the H1N1 pandemic vaccine strain (A/California/07/2009) and scored for divergence based on the percentage of amino acids that were similar at each position. The resulting clusters were then separated and non-pandemic strains – those with a divergence greater than the dashed grey line – were excluded from further analysis. (GIF) [file pone.0093632.s002.gif]

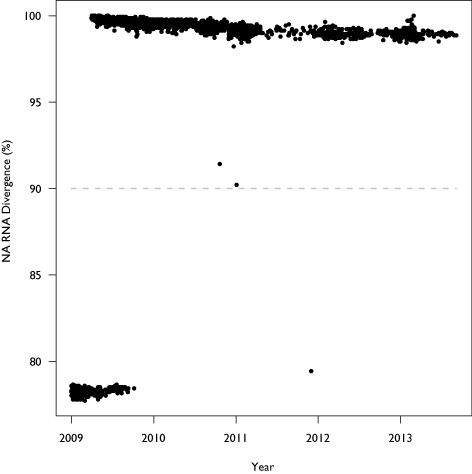

Supplement: Figure S3 — Influenza A(H1N1)pdm09 Strain Selection, Neuraminidase RNA. All H1N1 RNA coding sequences from January 1, 2009 to September 30, 2013 were compared to the H1N1 pandemic vaccine strain (A/California/07/2009) and scored for divergence based on the percentage of nucleotides that were similar at each position. The resulting clusters were then separated and non-pandemic strains – those with a divergence greater than the dashed grey line – were excluded from further analysis. (GIF) [file pone.0093632.s003.gif]

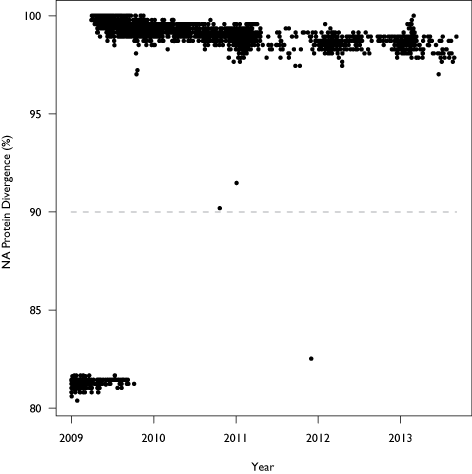

Supplement: Figure S4 — Influenza A(H1N1)pdm09 Strain Selection, Neuraminidase Protein. All H1N1 protein sequences from January 1, 2009 to September 30, 2013 were compared to the H1N1 pandemic vaccine strain (A/California/07/2009) and scored for divergence based on the percentage of amino acids that were similar at each position. The resulting clusters were then separated and non-pandemic strains – those with a divergence greater than the dashed grey line – were excluded from further analysis. (GIF) [file pone.0093632.s004.gif]

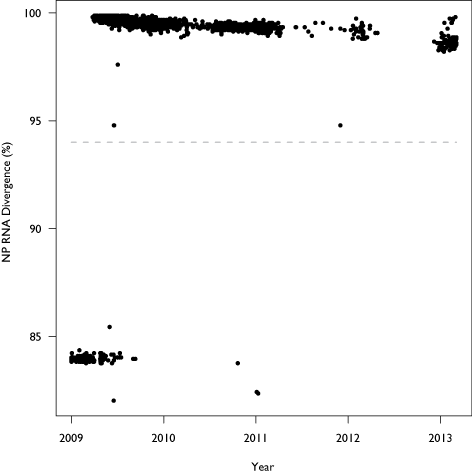

Supplement: Figure S5 — Influenza A(H1N1)pdm09 Strain Selection, Nucleoprotein RNA. All H1N1 RNA coding sequences from January 1, 2009 to September 30, 2013 were compared to the H1N1 pandemic vaccine strain (A/California/07/2009) and scored for divergence based on the percentage of nucleotides that were similar at each position. The resulting clusters were then separated and non-pandemic strains – those with a divergence greater than the dashed grey line – were excluded from further analysis. (GIF) [file pone.0093632.s005.gif]

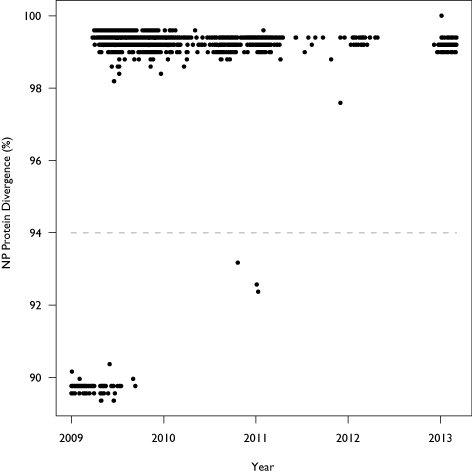

Supplement: Figure S6 — Influenza A(H1N1)pdm09 Strain Selection, Nucleoprotein Protein. All H1N1 protein sequences from January 1, 2009 to September 30, 2013 were compared to the H1N1 pandemic vaccine strain (A/California/07/2009) and scored for divergence based on the percentage of amino acids that were similar at each position. The resulting clusters were then separated and non-pandemic strains – those with a divergence greater than the dashed grey line – were excluded from further analysis. (GIF) [file pone.0093632.s006.gif]

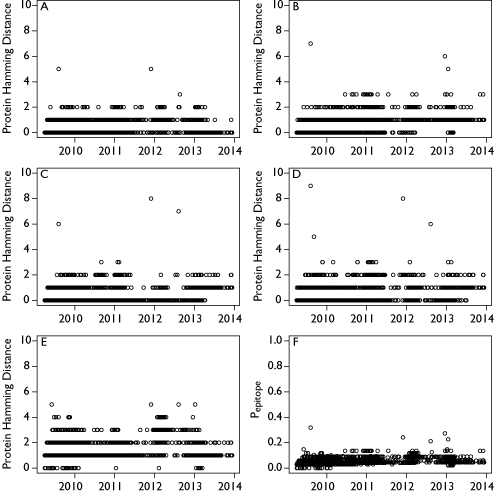

Supplement: Figure S7 — Divergence at the A(H1N1)pdm09 epitopes, definition 1. We used three potential descriptions of the epitope regions of the influenza A(H1N1)pdm09 HA protein. The present one was based on the A(H3N2) strain’s epitopes. A-E refers to the different epitopes, while F is the Pepitope calculation measuring the proportion of amino acid differences in the dominant epitope, for each strain. (GIF) [file pone.0093632.s007.gif]

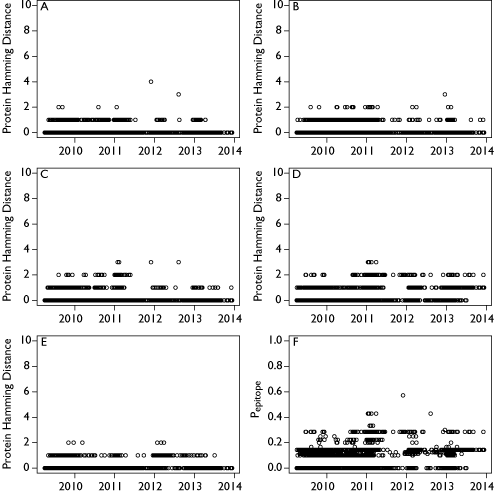

Supplement: Figure S8 — Divergence at the A(H1N1)pdm09 epitopes, definition 2. We used three potential descriptions of the epitope regions of the influenza A(H1N1)pdm09 HA protein. The present one is a set of natural epitopes that is a subset of the first set of epitopes. A-E refers to the different epitopes, while F is the Pepitope calculation measuring the proportion of amino acid differences in the dominant epitope, for each strain. (GIF) [file pone.0093632.s008.gif]

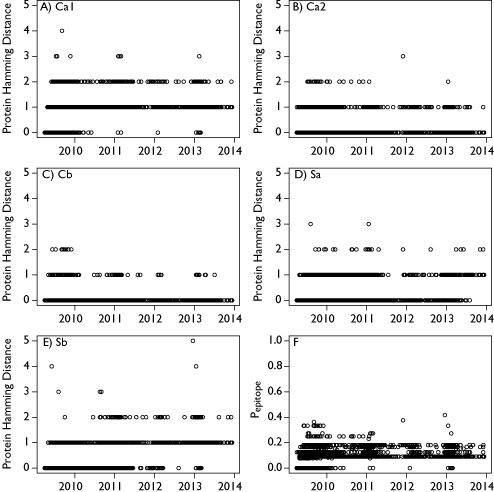

Supplement: Figure S9 — Divergence at the A(H1N1)pdm09 epitopes, definition 3. We used three potential descriptions of the epitope regions of the influenza A(H1N1)pdm09 HA protein. The present one is a set of laboratory confirmed epitopes for prior H1N1 strains. A-E refers to the different epitopes, Ca1, Ca2, Cb, Sa, Sb, while F is the Pepitope calculation measuring the proportion of amino acid differences in the dominant epitope, for each strain. (GIF) [file pone.0093632.s009.gif]

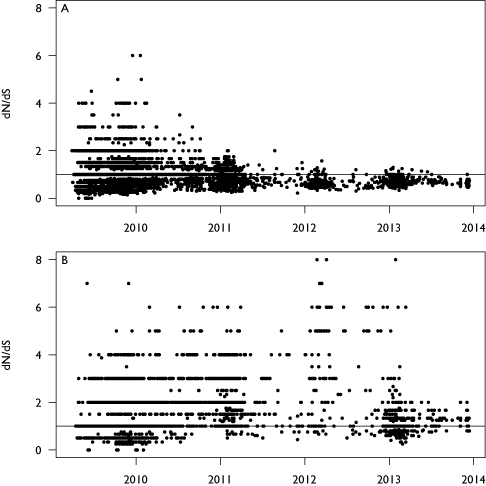

Supplement: Figure S10 — Non-Synonymous and Synonymous Mutations in A(H1N1)pdm09. We calculated the ratio of non-synonymous to synonymous mutations (dN/dS) for A(H1N1)pdm09 strains relative to the vaccine strain (A/California/07/2009) for regions outside the epitope regions (A) and within the epitope regions (B) using the first definition of the epitope regions (see methods). The straight line denotes unity, which is generally considered the neutral mutation rate. (GIF) [file pone.0093632.s010.gif]

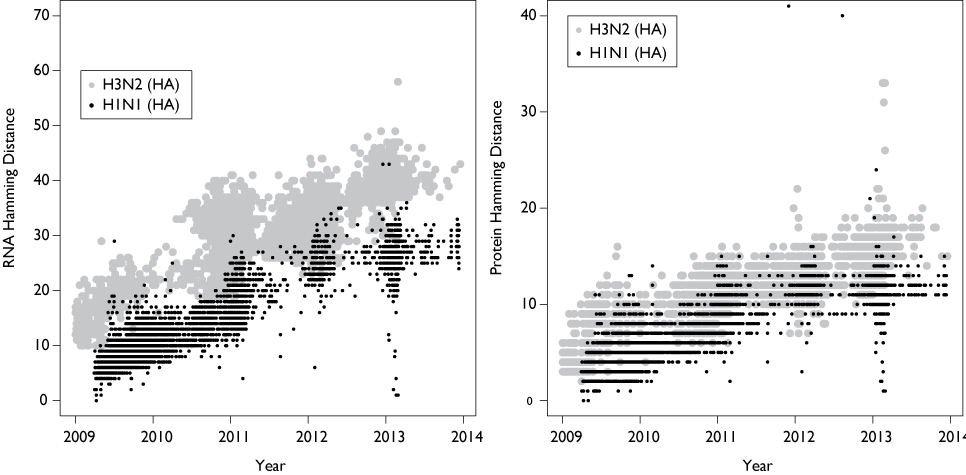

Supplement: Figure S11 — Comparison of A(H1N1)pdm09 and A(H3N2) evolution in the Hemagglutinin gene. We measured the hamming distance of the nucleotides (A) and the amino acids (B) relative to the vaccine strain for 2009. For H1N1 the vaccine strain (A/California/07/2009) has not changed since 2009, though it was not administered until November 2009. For H3N2 all isolates were compared to the A/Brisbane/10/2007 strain, though the vaccine has changed twice since then. While the hamming distance of H3N2 isolates from the vaccine strain continues to increase, the H1N1 isolates seem to have plateaued in recent years. (GIF) [file pone.0093632.s011.gif]
